# Distributed Camera Subsystem for Obstacle Detection

**DOI:** 10.3390/s22124588

**Published:** 2022-06-18

**Authors:** Petr Oščádal, Tomáš Spurný, Tomáš Kot, Stefan Grushko, Jiří Suder, Dominik Heczko, Petr Novák, Zdenko Bobovský

**Affiliations:** Department of Robotics, Faculty of Mechanical Engineering, VSB-TU Ostrava, 70800 Ostrava, Czech Republic; tomas.spurny@vsb.cz (T.S.); tomas.kot@vsb.cz (T.K.); stefan.grushko@vsb.cz (S.G.); jiri.suder@vsb.cz (J.S.); dominik.heczko@vsb.cz (D.H.); petr.novak@vsb.cz (P.N.)

**Keywords:** human–robot interaction, collaboration, workspace monitoring, distributed processing, sensors network, obstacles detection

## Abstract

This work focuses on improving a camera system for sensing a workspace in which dynamic obstacles need to be detected. The currently available state-of-the-art solution (MoveIt!) processes data in a centralized manner from cameras that have to be registered before the system starts. Our solution enables distributed data processing and dynamic change in the number of sensors at runtime. The distributed camera data processing is implemented using a dedicated control unit on which the filtering is performed by comparing the real and expected depth images. Measurements of the processing speed of all sensor data into a global voxel map were compared between the centralized system (MoveIt!) and the new distributed system as part of a performance benchmark. The distributed system is more flexible in terms of sensitivity to a number of cameras, better framerate stability and the possibility of changing the camera number on the go. The effects of voxel grid size and camera resolution were also compared during the benchmark, where the distributed system showed better results. Finally, the overhead of data transmission in the network was discussed where the distributed system is considerably more efficient. The decentralized system proves to be faster by 38.7% with one camera and 71.5% with four cameras.

## 1. Introduction

Increasing computing power allows more demanding tasks to be processed in less time. Despite this significant increase in computing power, the demands on the accuracy or volume of data processed are also increasing. Workspace monitoring is one example where such requirements grow even faster than computational power. There are multiple approaches to monitoring the environment. For example, depth measurement using time of flight [1,2,3] is not suitable for dynamic scenes. Structured light technology [4,5] is susceptible to ambient illumination. Passive sensing needs various textured objects. An extension for passive stereo vision offers an active approach [6], where a projector is added to a pair of cameras to project a pseudo-texture and enable better spatial sensing. Each has, of course, the positive aspects for which they were created and certain limitations. In general, several types of cameras or sensors can describe 3D environments and, nowadays, in great detail (millions of points) [7]. However, if a single sensor does not provide enough information, multi-camera systems can be used to combine information together [8]. It is necessary to think whether the whole environment needs to be captured in detail or focus should be given to a particular object. In any use of sensor systems, the primary focus is on the specific region of a workspace where the changes should be monitored in great detail. Thus, not all information obtained from sensors is relevant for subsequent use. It is almost always necessary to filter the input data to be satisfactory for the final product; for example, sensing people (namely for gaming using Kinect [9]), use in virtual reality, rehabilitation and similar situations from sensing people [10] to create 3D maps of the environment [11] requires specially designed filtering. It is possible to either filter static objects (the environment around people) or, on the contrary, filter known dynamic objects (e.g., a robotic arm). An important advantage is that it reduces the volume of data by removing information which is unnecessary for subsequent processing.

If we focus specifically on a workplace with a robot designed to collaborate with a human [12,13,14], it is required to monitor the workspace in which the robot can move without endangering the human.

Nowadays, many applications have been developed to improve Human–Robot Interaction (HRI) [15], which for example, use haptic feedback devices that notify a human worker about the currently planned trajectory and changes in the status of the robot [16,17]. An essential aspect of HRI is safety [18,19], in which robot avoidance against dynamic obstacles can be addressed using dynamically changing collision volumes [20,21], and alternatively, determining the robot’s speed based on detecting humans in the nearby space [22,23]. Nowadays, many applications are using neural networks [24,25] for detecting humans and predicting their movement [26,27].

**Figure 1:**
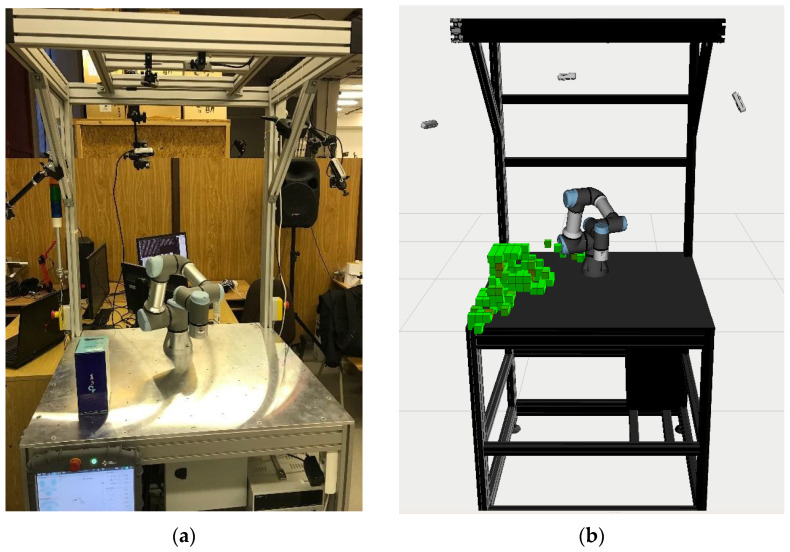
Demonstration image of the output from MoveIt! filtration: (**a**) real workspace; (**b**) simulation.

For such a situation, it is necessary to filter a static workplace and a moving robot within it. There are already functional tools for such an application, such as MoveIt! [28], implemented in the ROS (Robotic Operating System)[29] environment. MoveIt! allows connection of 3D cameras and the use of post-processing to filter the workplace data so that only obstacle information is retained, e.g., in the form of voxels [30] as illustrated in Figure 1 by the green obstacle voxels.

For testing or research, MoveIt! is one of the possible solutions for a quick implementation. This framework has an adjustable perception module for monitoring changes in the workspace of a robot. This perception pipeline works by firstly defining the cameras’ configuration files and then connecting the corresponding communication topics with the data from the cameras.

However, for industrial applications, this module has its own limitations. For example, there is a limitation on the speed of updating camera data, which has a huge impact on usability in real industrial applications. There can be multiple devices in a ROS system that are connected in a local network, which provides sensor data. This allows connecting any number of cameras without overloading the USB ports on one computer. On the other hand, more demands are placed on the local network, as the sensor data is not transmitted via USB but via Ethernet or Wi-Fi. In addition, the cameras that are used by the system must be defined when the system is started, which represents a major limitation as this severely limits the flexibility of the system in terms of a simple plug-and-play solution.

These limitations are due to the centralization of data processing from all cameras on one device.

The main contribution of this paper is a developed system to distribute process to separate units in the ROS structure. The communication is optimized so that the network is not overloaded (for processed data). Therefore, any unnecessary demands on the speed and structure of the network are not needed.

We present an easily implementable solution that enables a quick connection of multiple cameras to a system at runtime. Therefore, this system can be used for prototyping and the rapid reconfiguration of the workplace without restarting the entire monitoring subsystem, which makes it more flexible in terms of its ability to quickly adapt to various workplace-monitoring situations compared to conventional approaches.

## 2. Materials and Methods

**Figure 2:**
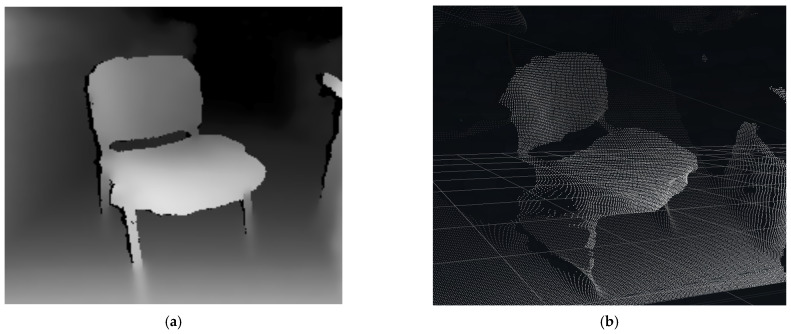
3D chair data: (**a**) depth image; (**b**) point cloud.

A fundamental aspect of 3D space representation is the form in which the environment is described. The most basic representation is the point cloud, which can be computed, for example, using stereo vision [7]. Based on the camera stream resolution, the number of points in space that correspond to each pixel in the depth image is obtained, see Figure 2.

**Figure 3:**
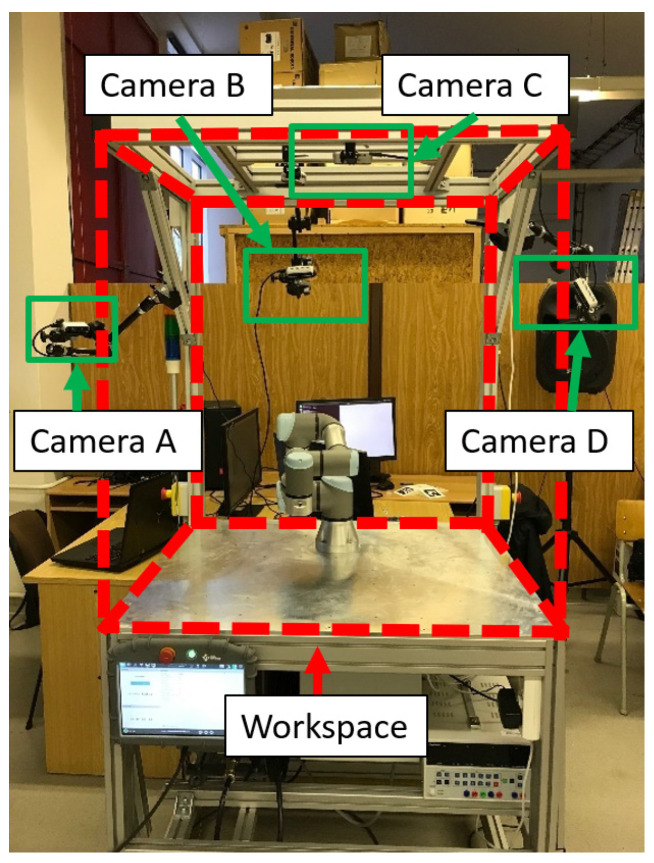
Workspace with robot UR3.

Capturing the entire space with a single camera can be problematic, as the camera only captures the surfaces of obstacles in front of it. Another problem with using just a single camera is overshadowing an object with a different object in front of it. To create a more accurate representation of the obstacle using a depth image, it is necessary to utilize multiple cameras in the space to capture the obstacles at different angles. Such systems are known as multi-camera systems. As an example of the multi-camera system, our workstation with four cameras monitors the workplace with a UR3 robot, see Figure 3.

With a multi-camera system, the workspace is scanned by multiple sensors, and the obtained data are then merged into a single representation. To fuse 3D data from multiple cameras, it is necessary to have a clearly defined camera position in space relative to a common base [31]. This can be achieved either by detecting visual markers [32] or by comparing point clouds relative to each other, from which the output is, for example, a transformation matrix. This matrix represents the rotation of the coordinate system around the *x*, *y*, and *z* axis and the displacement (1).

(1) $$CameraTransform=\left[\begin{array}{cccc} i_{x} & j_{x} & k_{x} & X\\ i_{y} & j_{y} & k_{y} & Y\\ i_{z} & j_{z} & k_{z} & Z\\ 0 & 0 & 0 & 1 \end{array}\right]$$

**Figure 4:**
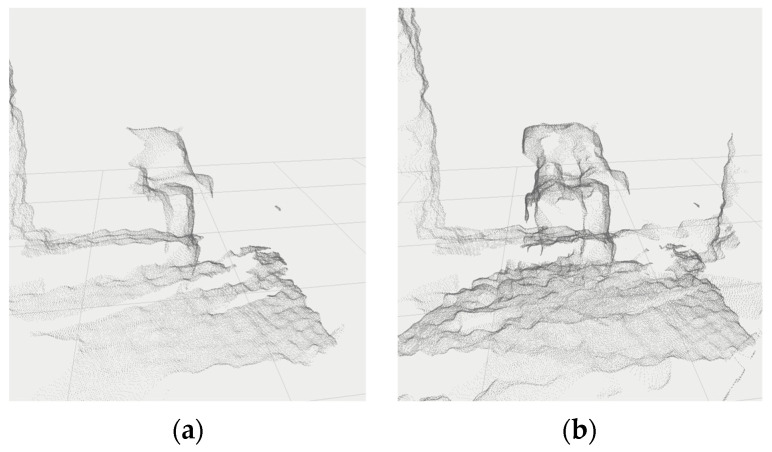
Merged point clouds (data from camera Intel Realsense D435i with 1280 × 720 resolution) in the workplace coordinate system: (**a**) data from camera D; (**b**) data from cameras A and D (cameras transformation was calibrated by [32]).

After the transformation, the individual point clouds are expressed relative to a single coordinate system and can be easily combined. The result is a single point cloud that describes the imaged workspace in more detail, Figure 4.

This way of representing the space can achieve a detailed description of the environment, but usually, such a detailed model is not needed, and the computational power for processing a large number of points grows enormously. Therefore, the data are simplified to an acceptable resolution by voxelizing the point cloud. Voxelization can be performed in several ways [30,33,34,35]. In our case, this is carried out by aligning it to a voxel-sized grid, see Algorithm 1.
**Algorithm1** Voxelization*voxels* = [] **for** *point* **in** *pointCloud*:  *voxel* = Voxel()  *voxel*.*x* = **Floor**(*point*.*x*/*voxelSize* + 0.5 * *voxelSize*)  *voxel*.*y* = **Floor**(*point*.*y*/*voxelSize* + 0.5 * *voxelSize*)  *voxel*.*z* = **Floor**(*point*.*z*/*voxelSize* + 0.5 * *voxelSize*)  *index* = *voxels*.**Exist**(*voxel*)  **if**(*index* ! = None):   *voxels*[*index*].*count* += 1  **else**:   *voxels*.**Append**(*voxel*)

**Figure 5:**
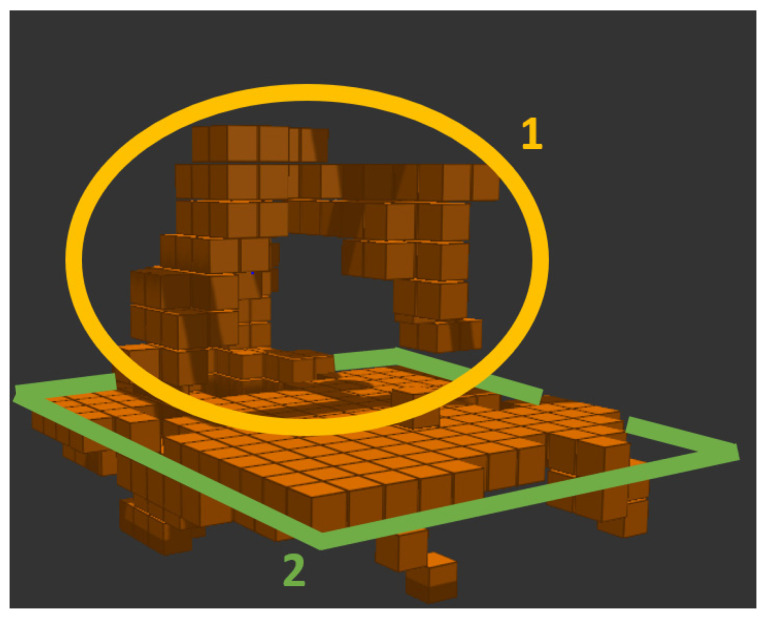
Cropping voxel map to workspace: 1. robot; 2. robot workspace boundaries.

The result of voxelization is a voxel map expressed by a point cloud that represents the volume of a grid-sized cube. The voxel size (which ranges from 10 to 100 mm in 10 mm increments) is the same throughout the entire image at all locations and can be changed in real-time. If the robot workspace should be captured, having 3D information about the distant surroundings is unnecessary. Therefore, the voxel map can be cropped to the maximum dimensions of the workspace and thus reduce the resulting number of points in the map, see Figure 5.

In this way, processed data are much more favorable for subsequent processing, although they still contain known objects. For example, the design of the workstation on which the robot is attached is clearly defined by the CAD model. This makes it unnecessary to capture this information and then reprocess it.

Therefore, filtering was implemented to filter the real data using an expected depth model. Expected depth can be obtained in two steps. First, the expected depth map is computed based on the perspective projection of 3D objects onto the 2D image, and then the expected depth for each pixel is computed, see Algorithm 2.
**Algorithm 2** Filter mask creation*depthImageExpected* = [] *models* = *database*.**GetActualScene**() **foreach** *mesh* **in** *models*:  *transformationMatrix* = *mesh*.*PositionMatrix* * *camera.ViewMatrix* * *camera.ProjectionMatrix*
 *mesh*.**Transform**(*transformationMatrix*)  *mesh*.**BackfaceCull**()  *mesh*.**ViewportScale**() *depthImageExpected*.**Draw**(*mesh*)

If the workplace is described by CAD models (in our case, STL models that represent the model by triangles), the first step is to transform the model into its actual position relative to the camera. Then, using a perspective projection matrix (Equation (5), which is composed of Equations (1)–(4)), the model is projected to the camera view. Where FOV represents the field of view, aspectRatio is the ratio between the width and height of the camera stream, nearPlane is the closest plane the camera can detect, and farPlane is the farthest plane where the camera can reconstruct depth.

The values in the perspective matrix were derived from the real Intel Realsense D435i camera. This camera uses active stereo vision. To improve detection, it uses an infrared projector for depth sensing. Cameras sensing the workstation do not interfere with each other, as the infrared map just supports better triangulation.

(2) $$yScale=\frac{1}{\tan\left(\frac{FOV}{2}\right)}$$

(3) $$xScale=\frac{yScale}{aspectRatio}$$

(4) $$planeScale=\frac{-nearPlane*farPlane}{farPlane-nearPlane}$$

(5) $$prespectiveMatrix=\left[\begin{array}{cccc} xScale & 0 & 0 & 0\\ 0 & yScale & 0 & 0\\ 0 & 0 & farPlane/\left(nearPlane-farPlane\right) & planeScale\;\\ 0 & 0 & 1 & 0 \end{array}\right]$$

To make the algorithm more efficient, all areas and faces of the 3D model that will be facing away from the camera view (hidden faces) were ignored. This is solved by checking the normals of the triangle area, see Algorithm 3.
**Algorithm 3** Backface culling**for** *triangle* **in** *mesh.Triangles*:  *vectAB* = *triangle*.*vertex* [1]–*triangle*.*vertex* [0] *vectAC* = *triangle*.*vertex* [2]–*triangle*.*vertex* [0] *normal* = **Cross**(*vectAB*, *vectAC*)  **if**(*normal.z* > 0):    *triangle*.*visible* = *false*

Since the model vertices are mapped (by projection matrix) to a range of <−1, 1> in both the X and Y axes, Algorithm 4 describes a general procedure to map triangle vertices to an arbitrary camera stream resolution, which, in turn, is dependent on the current depth stream setting.
**Algorithm 4** Viewport scale**for** *triangle* **in** *mesh.Triangles*:  **for** *vertex* **in** *triangle*:   *vertex*.*x* = int(*vertex*.x*wResolution/2) + *wResolution*/2   *vertex.y* = int(vertex.y*hResolution/2) + *hResolution*/2

**Figure 6:**
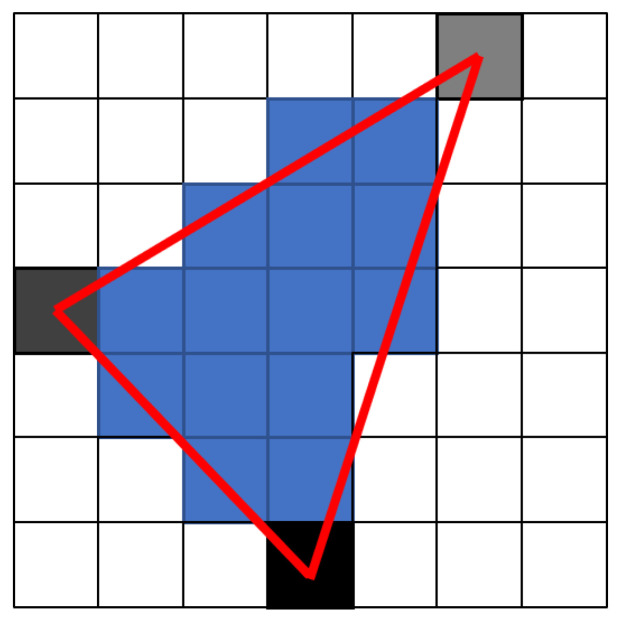
Triangle depth rasterizing.

At this point, there are already specific pixels of triangle vertices with expected depth. The depth represented by the greyscale of each vertex of the triangle is then interpolated for pixels inside the triangle (blue), see Figure 6.

In this way, all the visible faces (triangles) of the objects wanted to project into the depth image are drawn. There are also objects on the workstation described by CAD models but dependent on the current configuration, such as a robot. These objects need to be reconstructed from the current joint variables. Standard robots are described using Denavit–Hartenberg (DH) parameters that represent the relationship between the coordinate systems based on the current joint rotation [36]. Therefore, the actual matrices of the individual robot link displacements at the workstation must be derived sequentially. The transformation matrix for each joint is shown in Algorithm 5.
**Algorithm 5** Transformation matrix of individual robot elements**for** *i* **in** range(6):  $M_{i}=M_{i-1}*\left[\begin{array}{cccc} \cos\left(\vartheta_{i}\right) & -\sin\left(\vartheta_{i}\right)*\cos\left(\alpha_{i}\right) & \sin\left(\vartheta_{i}\right)*\sin\left(\alpha_{i}\right) & a_{i}*\cos\left(\vartheta_{i}\right)\\ \sin\left(\vartheta_{i}\right) & \cos\left(\vartheta_{i}\right)*\cos\left(\alpha_{i}\right) & -\cos\left(\vartheta_{i}\right)*\sin\left(\alpha_{i}\right) & a_{i}*\sin\left(\vartheta_{i}\right)\\ 0 & \sin\left(\alpha_{i}\right) & \cos\left(\alpha_{i}\right) & d_{i}\\ 0 & 0 & 0 & 1 \end{array}\right]$
where *ϑ*_i_ represents rotation around the *Z_i_*_−1_ axis (joint variable), *d*_1_ represents translation along the *Z_i_*_−1_ axis, *a*_i_ represents translation along the *X_i_*_−1_ axis, and *α*_i_ represents rotation around the *X_i_* axis, i.e., those kinematic parameters follow standard Denavit–Hartenberg convention.

**Figure 7:**
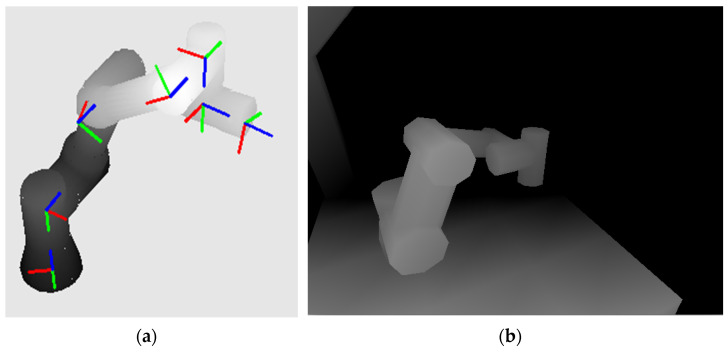
Rendered UR3 robot: (**a**) with coordinate systems attached to individual links according to the Denavit–Hartenberg principle (the blue *z-*axes represent the axes of rotation); (**b**) reconstructed expected depth map of the workspace.

The result is a reconstructed robot model according to the actual joint rotations, see Figure 7a. In Figure 7b, the result of an expected depth image created from the CAD models of the workspace and the actual position of the UR3 robot is shown.

**Figure 8:**
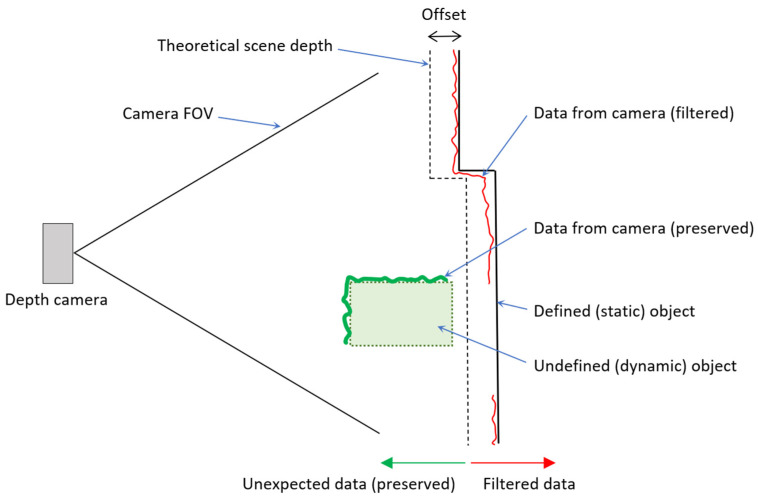
Illustration of filtration principle.

Once the actual scene has been completely reconstructed (Defined static/dynamic object), the real workspace scene obtained by the cameras can be compared with the calculated expected depth image. However, it is necessary to realize that the real camera captures with certain accuracy (data from the camera), and it is impossible to compare the real and expected depth exactly. Therefore, a sufficient offset needs to be added to cover the inaccuracy of the camera sensing, see Figure 8. This approach filters out all objects where the distance of expected depth (with offset) is less than the distance of the actual depth. The diagram shows the principle where the environment is defined (as a line Defined (static) object). The expected depth of the scene is identical to the real one but is offset to capture the basic inaccuracy of the camera (data from the camera(filtered)—red line). The un-defined (dynamic) object is before the expected depth (from the camera view), so this data is not filtered.

It is impossible to filter out all image noise by comparing the real and expected depth images. Therefore, was implemented a post-processing filter which determines whether a voxel is a noise voxel or an actual object surface based on the density of points in the voxel.

**Figure 9:**
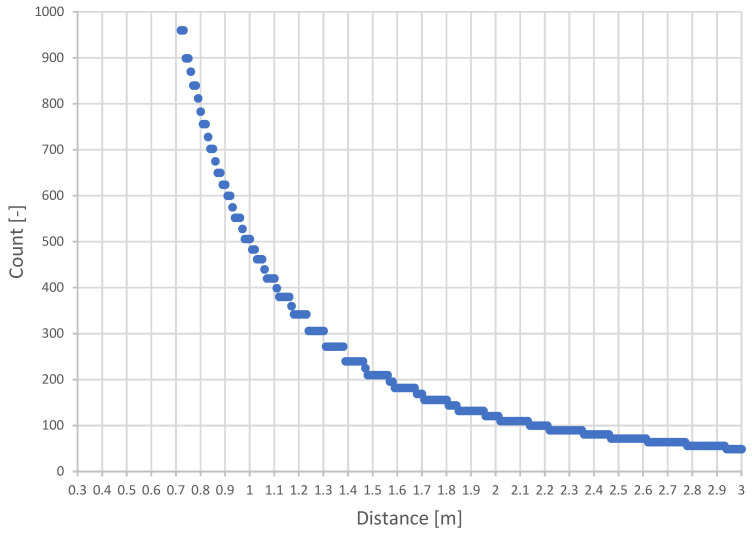
Density of expected points of a voxel depending on the distance from the camera.

The maximum possible density of points representing a voxel is variable based on the distance from the camera. The relationship between voxel occupancy and distance for a 5 cm voxel and the resolution of the Intel RealSense D435i 640 × 480 [px] camera is shown in Figure 9. It depicts the count of points per voxel according to the minimum and maximum possible sensing distance of the camera.

A simplified calculation of the maximum voxel capacity is described in Algorithm 6. First, the alpha angle is calculated, representing the maximum angle at which the rays of points can be projected. The density is then computed as the maximum number of rays that can fit into the alpha angle for pixels on the X and Y axis.
**Algorithm 6** Maximum number of points in a voxel*alfa* = **atan**(*voxelSize*/*voxel*.*distance*) *countPerRow* = *alfa*/(*hFOV*/*hResolution*) *countPerColumn* = *alfa*/(*wFOV*/*wResolution*) *countPerVoxel* = *countPerRow* * *countPerColumn*

The final filtering is then carried out by checking whether the actual voxel coverage is less than the density coverage with a certain threshold, and thus, these voxels are considered to be under-covered. Several factors affect the noise filtering threshold, e.g., ambient effects (sunlight) or camera lens calibration. In our case, this limit was around 50–60% of the maximum value to ensure that the filtering provides a satisfactory result. Hence, these voxels are removed as they represent noise, see Algorithm 7.
**Algorithm 7** Voxel filtering by occupancy percentage**for** *voxel* **in** *voxels*:  *maxCount* = **GetExpectedCount**(*voxel*.*Size*, *voxel*.*Distance*, *camera*.*Info*)  **if**(*voxel*.*count* < *maxCount* * *threshold*)   *voxels*.**Delete**(*voxel*)

The output of these algorithms are only voxels representing objects that have not been defined at the workplace and thus are needed for subsequent processing, e.g., robot trajectory re-planning.

**Figure 10:**
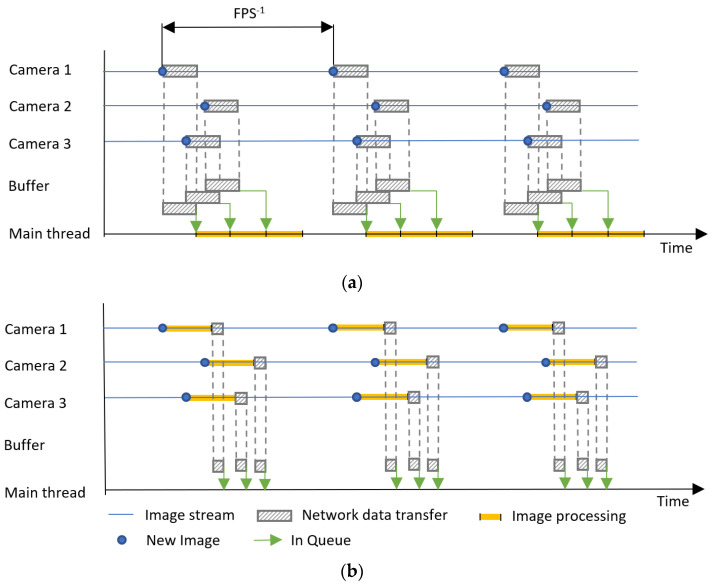
Diagram of the system principle: (**a**) centralized; (**b**) distributed.

The entire filtering process is distributed to individual computing devices that process depth streams from just one camera, as shown in Figure 10. Each of these units filters the scene for a specific camera position on the site. For our system, Nvidia Jetson Nano and Intel RealSense D435i cameras were used.

Figure 10 represents only symbolic block sizes and demonstrates the principle of a centralized and distributed system and the ratios do not correspond in any way to the real scenario. Figure 10 describes the case with three cameras at a general FPS (Frame Per Second).

In the initial setup of the cameras, all relative positions of the cameras to the robot coordinate system were obtained using a calibration grid board [27]. The system uses a single unit as the main camera, creating a server for initialization and a subsequent location for the descendant data, see Algorithm 8.
**Algorithm 8** Methods for Initialize and Update computing devices**def** Initialize(*device*):  **if**(*initializedDevices*.**Exist**(*device*.*IP*):   **return** *initializedDevice*[*device*.*IP*].*ID* *id* = *initializeDevices*.**Insert**(*device*.*IP*)  **return** *id* **def** Update(*device*):  *initializedDevices*[*device*.*ID*].*voxels* = *device*.*voxels* **return** *actualSceneValues*

The processed data from the individual units are combined in the main unit. Each computed datum is stored with a timestamp of when it was received to check for old data, see Algorithm 9. The verification is performed by comparing the time since the update of the data from the individual cameras. In other words, if the difference between the time of the arrival of data *T_U_* and actual time *T_A_* is less than *Threshold* (Equation (6)), the **IsTimeStampValid** condition is satisfied.

(6) $$T_{A}-T_{U}\;\leq \;Threshold\;$$

The units are not synchronized in the sense of camera frame acquisition. Each unit maintains its own framerate, and the most recent frame is sent to the master device, where it is used until a newer frame arrives (or the timestamp check fails).

Latency of the data update callback was measured as the time required for data transfer to the main device and transfer of the response data. The average transfer latency for 0 to 1000 voxels is 12 ms for each device (measured for four connected devices).
**Algorithm 9** Merging data from computing devices*globalVoxelMap* = [] **for** *device* **in** *initializedDevices*:  **if**(*device*.**IsTimeStampValid**())):   *globalVoxelMap*.**Insert**(*device*.*voxels*)

The Initialize method on the server represents the entry point for the processing units. When an initialization command arrives here, the incoming IP address is checked to see if it is already registered (behind one IP is expected one camera only). If it is registered, it represents a computing device that has been initialized in the past, and it is assumed that the device has been restarted either intentionally or due to a failure. No new memory space is created for such a computing device, and only its assigned ID is retrieved. The current workstation settings (e.g., robot position, filter parameters) are added to the ID. If the device IP is initialized for the first time, memory space is created that represents only the data from that unit. Allocated memory is represented by a dynamic list, and its initial size is for 150 voxels to avoid frequent increases in allocated memory. The output method then comprises the memory ID and following values, which represent the current settings (voxel size, robot configuration, percentage voxel occupancy, etc.), as is the case in pre-initialized devices.

The Update method is triggered if any computing unit updates the current data. The computing device that sends the data update also sends its ID. The server then deletes all previous data from that unit and replaces it with the new data. In addition, the timestamp of the newly arrived values is recorded so that the data can be checked to confirm whether the data is outdated for future data fusion.

When connected to the network, each computing device must initialize itself to obtain an ID under which it will then send the current local data it is currently capturing with the connected camera. A localization algorithm (in our case, 3D grid board detection) is used to find the position of the camera connected to a particular device. The depth map is then filtered using the 3D reconstruction of the site and the depth map comparison described above.

Using this structure, any number of computation devices with a camera (Jetson Nano; Nvidia, Santa Clara, CA, USA and D435i camera; Intel, Santa Clara, CA, USA) can be connected. Furthermore, the module is set up to run the filtering algorithms when the system starts automatically, so there is no need to have a device (e.g., keyboard, mouse, monitor, etc.) connected to each unit, and it will automatically start streaming the current data when the power is turned on.

**Figure 11:**
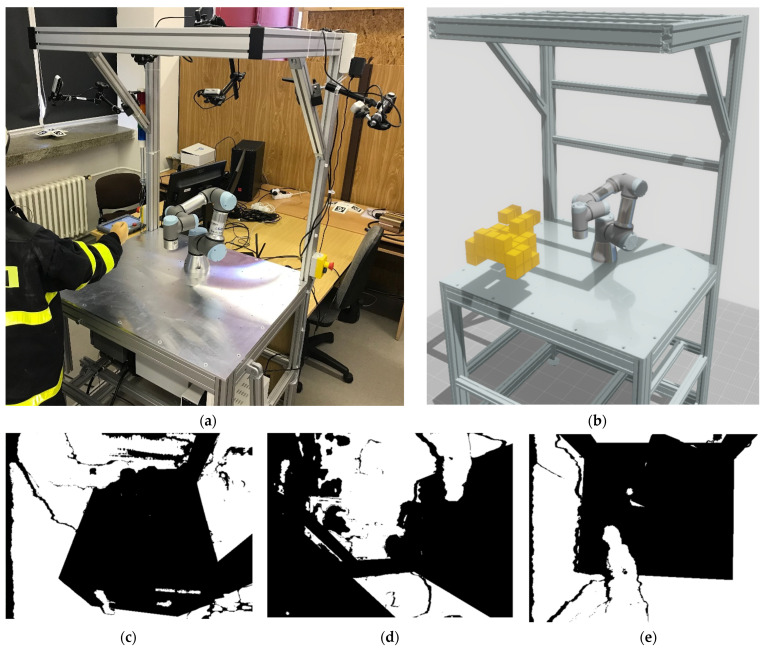
Filtered scene by the distributed method: (**a**) real scenario; (**b**) 3D visualization; (**c**–**e**) camera bitmask.

Based on the idea of distributing the computational power, algorithms have been proposed to filter the depth information in the dynamic environment (Figure 11a). This information was then thoroughly filtered (Figure 11c–e) so that the output data from the sensor system contained only the necessary information about unknown objects in the scene, see Figure 11b.

## 3. Results and Discussion

The depth image processing speed and refresh rate of the entire workplace scene were compared using the distributed method versus the centralized (MoveIt!) method in a real workplace. It should be noted that the depth image filtering was performed differently by each method. In the distributed method, the computation was performed on the Jetson Nano unit processor (Quad-core ARM A57, 1.43 GHz, Memory 4 GB LPDDR4) [37], while in the centralized method, everything was computed on the laptop GPU (NVIDIA GeForce GTX 1070, 1.51 GB, CUDA Cores 1920, Memory 5 GB GDDR5) [38].

Variables such as the resolution of cameras, the size of the voxel map, and the number of cameras were used to compare the solutions. These factors affect the total performance of the solution with a major impact on the scene refresh rate, hence the need for measurements.

The scene refresh rate of the workstation was measured for the following depth stream resolutions with a setting of 15 FPS:

1. 424 × 240;
2. 640 × 480;
3. 1280 × 720.

For all of these resolutions, the voxel grid size dependencies were measured, where the voxel size was measured from 10 to 100 mm in 10 mm increments. The actual measurement consisted of determining the time to compute the depth image from the time of its arrival to the filtering and the time to reconstruct the entire workstation scene from the reception of the data from the first camera to the evaluation of the filtering from the last camera. Table 1 and Table 2 compare the time for reconstructing the whole scene with three cameras, using both the centralized and distributed approaches. During the measurements, it was found that the centralized approach at higher resolutions was not able to provide a depth stream of 15 fps. At a resolution of 424 × 240, the framerate was maintained. At 640 × 480 resolution, the depth stream reached a maximum of 6 fps, and at 1280 × 720, it reached 2 fps. A low bandwidth of the depth stream then influences the processing time of the image. Furthermore, it was found that when the voxel size was less than 0.02 m, the depth filtering time increased rapidly: for 424 × 240 resolution, the time increased to 1.9 s; for 640 × 480 resolution, the time increased to 2.0 s, and for 1280 × 720 resolution, it increased to 2.9 s (Table 1). This problem could be, for example, caused by memory management. At lower voxel resolutions, multiple memory uses occur, thus redistributing the initial reserved space. This substantially increases the processing time for the entire scene. On the other hand, there was no problem maintaining the required 15 fps even with 1280 × 720 resolution for the distributed approach. The scene refresh rate ranges from 0.05–0.3 s across all measured resolutions for the distributed method.

All processed values across resolutions are listed in Table 1 for the centralized system and Table 2 for the distributed system. For a better comparison, the percentage value of the average difference was added to Table 2, which is calculated by Equation (7):

(7) $$Avg_{diff}=100-\frac{Avg_{D}}{Avg_{C}}\cdot 100$$

where $Avg_{D}$ is required average time to process the whole scene using the decentralized approach and $Avg_{C}$ using the centralized approach.

**Figure 12:**
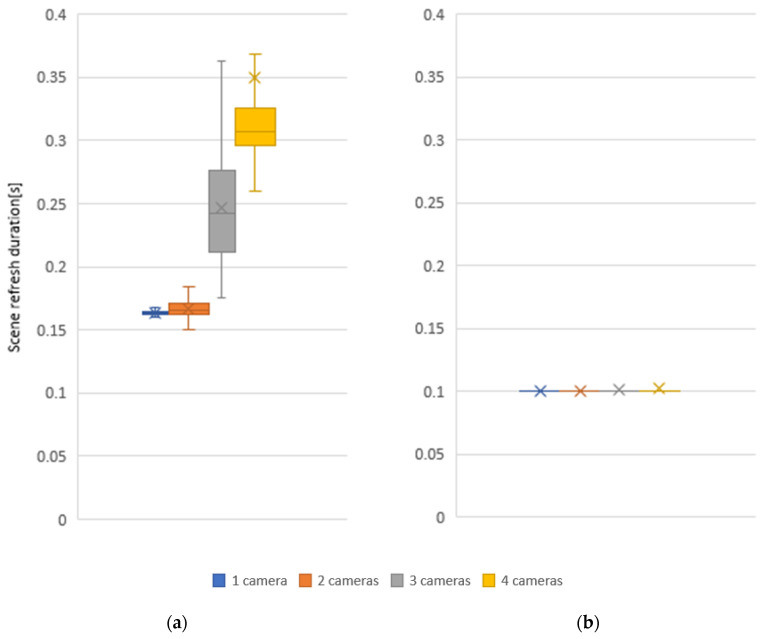
Effect of the number of cameras on the scene refresh duration for 1280 × 720 camera resolution and voxel size 20 mm: (**a**) centralized; (**b**) distributed.

Subsequently, the effect of the number of cameras on scene refresh rate was measured for one, two, three, and four cameras in the workplace. The centralized system processes the camera data serially and does not allow the connecting of multiple cameras for a more detailed real-time mapping of the whole space since as the number of cameras increases, the time to recover the whole scene increases. In a distributed system, this problem does not arise since the computation is performed in parallel and is therefore not dependent on the number of cameras, see Figure 12.

Scene refresh rate measurements based on the number of cameras have clearly shown that a system that filters data from each camera separately and sends the resulting data to the main device where all data are combined into a global voxel map is more efficient to use. The distributed system refresh rate is faster by 38.7% when using one camera, by 40.1% when using two cameras, 59.4% when using three cameras, and 71.5% when using four cameras. This ensures a more stable refresh rate of the whole scene even with larger numbers of sensors and reduces the network requirements as it does not need to send source data from cameras between devices.

Data sent over the network within a centralized system is sent in raw format (depth image), which equals the image width times height times the bit rate of the depth stream for each camera in the system. For example, when capturing at 640 × 480 resolution and a 32-bit depth image, then 9.8 Mbit of data needs to be transferred. For three cameras, this is therefore 29.4 Mbit/scene refresh. While in a distributed system, the data sent is based on the current scenario (nothing is sent if there are no undefined objects). When sending voxels (either an index in the grid or specific X,Y,Z coordinates), one can count 3 × 32 bits per voxel, whereas, in a normal scenario, the unit sends less than 100 voxels on average. Then, on average, 9.3 Kbit per camera can be counted, and with three cameras, it is only 27.9 Kbit.

To make a distributed system less efficient, the number of voxels sent would have to be higher than 1/3 of the number of pixels which is very unlikely with the current filtering.

Needed time may also be compared for implementing the system. In the case of a centralized system (MoveIt!), it was needed to manually change parts of the code responsible for placing cameras into the workspace (if no additional script was created for conducting this routine instead of us). That means if there was a need to change the number of cameras, there was a need to make changes in the code. On the other hand, while using a distributed system, it is needed just to place or remove a camera from the system with its computation unit to the change number of cameras. This makes this plug-and-play solution really user-friendly and much faster to implement. There is also an advantage in the case of system reachability. With a centralized system, reachability is limited by the length of the camera’s USB cable (which is around 2 m for securing fast data transfer) if only one computer is used. However, with distributed system, a 2 m USB cable should be always enough to reach the computing unit. Communication between the main device and computing units is addressed by a high-speed ethernet cable. This means there is a possibility of a greater spread of cameras in the workspace in the case of a distributed system.

## 4. Conclusions

Workspace monitoring is one of the essential elements of a workplace where a human has to interact with a robot. Reconstructing dynamic objects in the workplace can ensure safety for the operator and smooth operation of the workplace because the robot can react to changes in the free space for movement. The workplace reconstruction process is standard through packages in the Robotic Operation System (ROS). These packages are designed for universal use. However, this has the effect of centralizing the computing power into a single device. Such a solution is less suitable for processing large volumes of data from multiple sensors because the user does not have complete control over the computational processes.

Therefore, a new principle has been proposed that distributes the computational power among the individual devices. Each processing device allows filtering of the locally sensed data. The filtering is based on a 3D description of the workplace and the creation of a density camera depth map for each camera separately from their point of view. Filtering compares the actual depth map from the sensor and the calculated density depth map. The resulting data are information about undefined obstacles (arms, boxes, etc.). These obstacles are subsequently processed by noise filtering. The resulting data from the individual distributed devices are combined in the main computer (Algorithm 9), which produces an overall description of the entire sensed workspace.

This distributed sensing method was then verified and compared with the currently available centralized system (MoveIt!). The results show that both solutions are comparable across camera setups for different resolutions and voxel map sizes, but the main advantages of the distributed system are in the stable refresh rate of the whole scene when the number of sensors involved in the system changes. The measurements showed that when the number of cameras increased (1–4 cameras were measured), the scene refresh rate of the centralized system decreased substantially. In contrast, the distributed system maintained its scene refresh rate. Despite the fact that in a distributed system, the computation is performed in parallel on individual devices, when merging the data, there is a timestamp check to avoid unwanted effects (ghosts, broken data, etc.).

Based on the measurements, the system had shown to be able to maintain the workspace sensing rate even when the sensing parameters change. The essential parameter is the number of cameras, which significantly influences the refresh rate of the entire scene. The assumption that the distributed system is independent of the number of cameras has been proven, compared to the centralized system, which is slower with each additional camera added to the system. This assumption is especially evident when processing data from more than three cameras, where the data calculation from the whole scene is almost constant. If security in a given area should be ensured, the number of cameras will not be a limiting factor.

In terms of hardware, the only difference is in the final computing unit, which has higher computing power requirements in a centralized system than in a distributed system. The goal of this work was not to determine the exact computational power required for each system. The hardware in this work was selected based on previous experiences.

Despite the fact that filtering on distributed units runs on the CPU, the speed is comparable to the currently available solution in MoveIt!. Of course, this solution can be adapted to compute using a GPU, which can increase the speed; in general, this can be used on units that have graphic power available.

Future work could focus on noise elimination. In the current solution, voxel occupancy is judged based on the percentage of 3D points from a single camera. This approach could be more robust when using information from multiple cameras (e.g., two cameras detect a voxel as empty, and a third camera detects it as occupied). Further, the functionality of the system could be extended to automatically assign a size to each voxel based on the size and shape of the detected feature. This could lead to a reduction in the size of the total number of detected voxels. Consequently, it would be desirable to examine the trend of the ability to efficiently merge the processed camera image data. It is assumed that when processing tens of cameras (30–50 cameras), the rate of data merging should be constantly the same. This number of used cameras could be possibly used in the case of a huge workspace. However, the use of a higher number of cameras in the measurements was not considered, due to the insufficient budget to purchase a large number of cameras.

## Figures and Tables

**Table 1:** Time required to process the whole scene with three cameras for various voxel sizes and camera resolutions using the centralized approach.

Voxel Size (m)		0.01	0.02	0.03	0.04	0.05	0.06	0.07	0.08	0.09	0.1
424 × 240	Min (ms)	1303	146	47	34	18	22	15	24	16	18
	Max (ms)	16,867	944	163	121	149	152	138	145	149	143
	Median (ms)	1909	214	74	67	67	67	67	67	67	67
	Average (ms)	1960	217	78	67	67	67	67	67	67	67
640 × 480	Min (ms)	1305	146	71	123	145	133	155	152	135	120
	Max (ms)	11,531	997	224	476	202	210	202	205	203	190
	Median (ms)	2007	234	168	167	166	166	166	166	166	166
	Average (ms)	2087	248	169	172	166	167	166	168	167	167
1280 × 720	Min (ms)	2031	339	419	409	341	255	423	410	247	368
	Max (ms)	21,894	616	609	561	565	555	559	554	582	560
	Median (ms)	2416	504	493	492	503	500	495	490	490	495
	Average (ms)	2981	503	496	494	501	496	496	491	491	495

**Table 2:** Time required to process the whole scene with three cameras for various voxel sizes and camera resolutions using the distributed approach. For each camera resolution, the percentage difference of the average value (Avg. diff) compared to the centralized system is shown.

Voxel Size (m)		0.01	0.02	0.03	0.04	0.05	0.06	0.07	0.08	0.09	0.1
424 × 240	Min (ms)	37	37	38	38	37	37	37	37	37	37
	Max (ms)	63	63	63	63	63	63	62	63	63	63
	Median (ms)	50	50	50	50	50	50	50	50	50	50
	Average (ms)	50	50	50	50	50	50	50	50	50	50
	Avg. diff (%)	**97.45**	**76.96**	**35.90**	**25.37**	**25.37**	**25.37**	**25.37**	**25.37**	**25.37**	**25.37**
640 × 480	Min (ms)	98	98	98	87	97	87	88	91	93	87
	Max (ms)	114	115	115	126	119	125	125	115	115	163
	Median (ms)	100	100	100	100	100	100	100	100	100	100
	Average (ms)	104	104	104	104	104	104	104	104	104	104
	Avg. diff (%)	**95.02**	**58.06**	**38.46**	**39.53**	**37.35**	**37.72**	**37.35**	**38.10**	**37.72**	**37.72**
1280 × 720	Min (ms)	275	279	284	275	286	237	274	286	274	274
	Max (ms)	305	300	301	307	301	301	325	313	300	325
	Median (ms)	287	288	288	288	288	288	287	288	287	287
	Average (ms)	287	288	290	288	290	289	287	292	286	287
	Avg. diff (%)	**90.37**	**42.74**	**41.53**	**41.70**	**42.12**	**41.73**	**42.14**	**40.53**	**41.75**	**42.02**

## Data Availability

The data presented in this study are available on request from the corresponding author. The data are not publicly available due to project restrictions.
